# The role and interaction of imprinted genes in human fetal growth

**DOI:** 10.1098/rstb.2014.0074

**Published:** 2015-03-05

**Authors:** Gudrun E. Moore, Miho Ishida, Charalambos Demetriou, Lara Al-Olabi, Lydia J. Leon, Anna C. Thomas, Sayeda Abu-Amero, Jennifer M. Frost, Jaime L. Stafford, Yao Chaoqun, Andrew J. Duncan, Rachel Baigel, Marina Brimioulle, Isabel Iglesias-Platas, Sophia Apostolidou, Reena Aggarwal, John C. Whittaker, Argyro Syngelaki, Kypros H. Nicolaides, Lesley Regan, David Monk, Philip Stanier

**Affiliations:** 1 Genetics and Epigenetics in Health and Diseases Section, Genetics and Genomic Medicine Programme, UCL Institute of Child Health, London WC1N 1EH, UK; 2 Noncommunicable Disease Epidemiology Unit, London School of Hygiene and Tropical Medicine, University of London, London WC1E 7HT, UK; 3 Harris Birthright Research Centre for Fetal Medicine, King's College Hospital, London SE5 9RS, UK; 4 Department of Obstetrics and Gynaecology, Imperial College London, St Mary's Campus, London W2 1NY, UK

**Keywords:** genomic imprinting, fetal growth restriction, placenta, chorionic villus sampling, birth weight, type 1 diabetes

## Abstract

Identifying the genetic input for fetal growth will help to understand common, serious complications of pregnancy such as fetal growth restriction. Genomic imprinting is an epigenetic process that silences one parental allele, resulting in monoallelic expression. Imprinted genes are important in mammalian fetal growth and development. Evidence has emerged showing that genes that are paternally expressed promote fetal growth, whereas maternally expressed genes suppress growth. We have assessed whether the expression levels of key imprinted genes correlate with fetal growth parameters during pregnancy, either early in gestation, using chorionic villus samples (CVS), or in term placenta. We have found that the expression of paternally expressing insulin-like growth factor 2 (*IGF2*), its receptor *IGF2R*, and the *IGF2*/*IGF1R* ratio in CVS tissues significantly correlate with crown–rump length and birthweight, whereas term placenta expression shows no correlation. For the maternally expressing pleckstrin homology-like domain family A, member 2 (*PHLDA2*), there is no correlation early in pregnancy in CVS but a highly significant negative relationship in term placenta. Analysis of the control of imprinted expression of *PHLDA2* gave rise to a maternally and compounded grand-maternally controlled genetic effect with a birthweight increase of 93/155 g, respectively, when one copy of the *PHLDA2* promoter variant is inherited. Expression of the growth factor receptor-bound protein 10 (*GRB10*) in term placenta is significantly negatively correlated with head circumference. Analysis of the paternally expressing delta-like 1 homologue (*DLK1*) shows that the paternal transmission of type 1 diabetes protective G allele of rs941576 single nucleotide polymorphism (SNP) results in significantly reduced birth weight (−132 g). In conclusion, we have found that the expression of key imprinted genes show a strong correlation with fetal growth and that for both genetic and genomics data analyses, it is important not to overlook parent-of-origin effects.

## 1. Background and results

### (a) Fetal growth

Birthweight and its relationship to mortality show one of the strongest links observed in epidemiology, illustrated by a reverse-J-shaped curve with the highest mortality observed in the lightest and heaviest groups [1]. Growing appropriately *in utero* is essential for a long and healthy life. Fetal growth restriction (FGR) affects approximately 6% of pregnancies, and is identified in approximately half of stillborn fetuses without malformations [2,3]. While the majority of FGR babies demonstrate catch-up growth, the combination of suboptimal intrauterine growth followed by accelerated childhood growth can increase their susceptibility to adult-onset diseases, including type 2 diabetes, hypertension and coronary artery disease [4]. Each baby's unique growth potential *in utero* is determined by the successful nutritional and respiratory support from the mother to the fetus via a placenta, and disturbing this balance could lead to FGR [5]. Fetal growth is influenced by both genetic and environmental factors, although the relevant molecular pathways are still poorly defined. Identifying key genes and pathways that regulate fetal growth will allow for better monitoring of intrauterine growth, maximizing healthy outcomes.

### (b) Genomic imprinting

Genomic imprinting is a process of epigenetic modification on the genome that causes silencing of one allele according to its parental origin, resulting in monoallelic expression, without changing the DNA sequence [6–8]. Sex-specific imprint marks are heritable to daughter cells, but are erased and re-established in the germline during gametogenesis [9]. Evidence from mouse models and rare human imprinting disorders suggests that genes that are paternally expressed tend to increase fetal growth, whereas maternally expressed genes restrict fetal growth. For example, mice knockouts for paternally expressed genes *Igf2*, mesoderm-specific transcript (*Mest*) and paternally expressed gene 3 (*Peg3*) result in FGR, whereas mice deficient for maternally expressed genes insulin-like growth factor 2 receptor (*Igf2r*), *H19* and *Grb10* show an overgrowth phenotype [10–14] (table 1). Rare imprinting disorders such as the growth-restricted phenotype of Silver–Russell syndrome (SRS) may implicate complex roles involving both absence of growth promoters such as *IGF2* and potential increase of growth restrictors such as *GBR10* (reviewed in [26]).

**Table 1.** Imprinted genes highly expressed in the placenta. Origin, parental origin of the expressed allele; M, maternally expressed; P, paternally expressed; ncRNA, non-coding RNA; FGR, fetal growth restriction; Dup, duplication; UPD, uniparental disomy; ICR, imprinting control region; LBW, low birthweight; BW, birthweight; HC, head circumference; CVS, chorionic villus sampling tissues; CRL, crown–rump length; PIP, phosphatidylinositol phosphate lipid; mat del, maternally inherited deletion; pat del, paternally inherited deletion; T1D, type 1 diabetes; TNDM, transient neonatal diabetes mellitus; BWS, Beckwith–Wiedemann syndrome; SRS, Silver–Russell syndrome; CNV, copy number variation; asterisk, findings from this study.

locus	gene	origin	description	mouse KO phenotypes	human growth phenotypes
6q24	*PLAGL1*	P	zinc finger protein	FGR, bone malformation, high neonatal lethality [15]	TNDM (pUPD6, pDup6q24, ICR hypomethylation) [16]
6q25	*IGF2R*	M/biallelic	clearance of IGF2	fetal and placental overgrowth, organ and skeletal abnormalities [11]	CVS expression positively correlated to BW [17] and CRL*
7p12	*GRB10*	M/P	GF receptor-bound protein	fetal and placental overgrowth [10]	implicated in SRS (mDup7p11.2–13) [18]; term placenta expression negatively associates with HC*
7q21.3	*PEG10*	P	retrotransposon derived	embryonic lethal due to placental malformation [19]	hypermethylation at ICR and reduced expression in LBW cord blood [20]; upregulated in FGR placenta [21]
7q32.2	*MEST*	P/biallelic	α/β hydrolase fold family	fetal and placental growth restriction, high postnatal lethality, abnormal maternal behaviour [12]	implicated in SRS (mUPD 7q31-qter) [22]
11p15	*H19*	M	long ncRNA	fetal and placental overgrowth [13,23]	ICR1 hypomethylation [24] and CNV [25] in SRS
	*IGF2*	P	growth factor	fetal and placental growth restriction	CVS expression positively correlated to BW [17] and CRL*; implicated in BWS and Wilm's tumour [26]
	*CDKN1C*	M	tumour suppressor	gestational fetal and placental overgrowth [27]	mutated in IMAGe [28], BWS [29] and SRS [30] patients
	*SLC22A18*	M	organic cation transporter	not reported	term placenta expression associated with HC [31]
	*PHLDA2*	M	PH domain, PIP binding	placental overgrowth [32]	highly expressed in lower BW and FGR placenta [21,33–35]; promoter variant associated with BW [36]
14q32	*DLK1*	P	transmembrane glycoprotein	pre- and postnatal growth restriction, high perinatal lethality, obese postnatally [37]	associated with T1D [38], UPD14 syndromes [26], T1D SNP correlated to BW*
	*MEG3*	M	ncRNA	postnatal lethal (mat del), pre- and postnatal growth restriction, high perinatal lethality (pat del) [39]	associated with T1D [38], reduced expression in FGR placenta [35]
19q13.4	*PEG3*	P	zinc finger protein	placental and fetal growth restriction, abnormal maternal behaviour [14]	tumour suppressor [40]

The kinship theory or parental ‘conflict theory’ predicts that imprinting may have evolved as a result of competition between the paternal and maternal genome for maternal nutrient provision. The paternal genome encourages fetal growth by extracting nutrients from the mother, whereas the maternal genome counterbalances this by limiting resources to the offspring to ensure not only her survival, but also the equal provision of nutrients among her offspring [41]. Genomic imprinting is observed predominantly in placental mammals, and it is, indeed, the placenta which serves as the key regulatory site for this genomic conflict.

More than 100 imprinted genes have been identified in mice and approximately half of them are conserved in humans. In addition to this, many more tissue-specific human-imprinted loci are being discovered (http://igc.otago.ac.nz/; http://www.har.mrc.ac.uk/) [42]. In the current project, we have studied 13 imprinted genes that are highly expressed in human term placenta and are known to lead to growth phenotypes when deficient in mice (table 1). In addition, we included three non-imprinted genes that were critical to the action of *IGF2*, which is a key paternally expressed imprinted growth promoter (table 2). We have investigated the expression of these genes in both early and late gestation using the King's College London (KCL) CVS cohort (11–13 weeks of gestation) and the Moore term placenta cohort, respectively, and correlated these data with important growth parameters such as birthweight, placental weight and head circumference.

**Table 2.** Non-imprinted genes highly expressed in the placenta.

locus	gene	description	mouse KO phenotypes	human growth phenotypes
7p12	*IGFBP3*	carrying protein for IGF1 and IGF2	retinal vessel loss [43]	implicated in common cancers [44]
12q23.2	*IGF1*	growth promoter	pre- and postnatal growth restriction, infertile [45]	pre- and postnatal growth restriction [46]
15q26.3	*IGF1R*	IGF1 and IGF2 receptor	fetal growth restriction and perinatal lethal	pre- and postnatal growth restriction [47]

Also, in a separate analysis reported here, the potential influence of other variables such as the baby's sex, gestational age, parity, maternal weight/body mass index (BMI) and maternal smoking were tested against gene expression. In some situations, loss of imprinting (LOI) can occur, leading to biallelic expression of the gene. Because this could potentially influence the overall gene dosage, term placenta and CVS samples used in these expression studies were also investigated to see whether they retained a normal imprinting pattern, or showed monoallelic expression. In this hybrid review/research article, we summarize our previous findings together with new data.

### (c) Insulin-like growth factor axis and *IGF2*/*H19* locus

The insulin/IGF growth factor ‘axis' constitutes key regulatory endocrine factors of pre- and postnatal growth. These include insulin (*INS*), *IGF1*, *IGF2* and their corresponding receptors (*IR*, *IGF1R* and *IGF2R*), and six binding proteins (*IGFBP1–6*) [48]. INS and IGF1 exclusively bind to IR and IGF1R, respectively, whereas IGF2 can bind to IGF1R, IGF2R and IR 11-isoform [49]. *IGF2R* is located on human chromosome 6q25.3 and shows maternal expression in only 10% of term placentas and CVS [17,50]. One of its major functions is the lysosomal targeting and degradation of IGF2, thus acting as a growth suppressor [51]. *IGF2* and *H19* map to one of the most intensely studied imprinted gene clusters on human chromosome 11p15. Their reciprocal imprinting is controlled by differential methylation of imprinting control region 1 (ICR1) which is normally only methylated on the paternal allele [52]. The unmethylated maternal ICR1 allows the binding of the CTCF transcription factor, blocking the access of *IGF2* promoters to the *H19* downstream enhancers, resulting in the activation of *H19* expression. Conversely, the CTCF protein is prevented from binding to the paternal methylated ICR1, resulting in monoallelic paternal *IGF2* expression owing to *IGF2* promoter interaction with the enhancers. Approximately 50% of the growth-restricted SRS cases show loss of methylation at ICR1, which could lead to decreased *IGF2* expression [24] and that may well contribute to SRS growth restriction.

**Figure 1.**
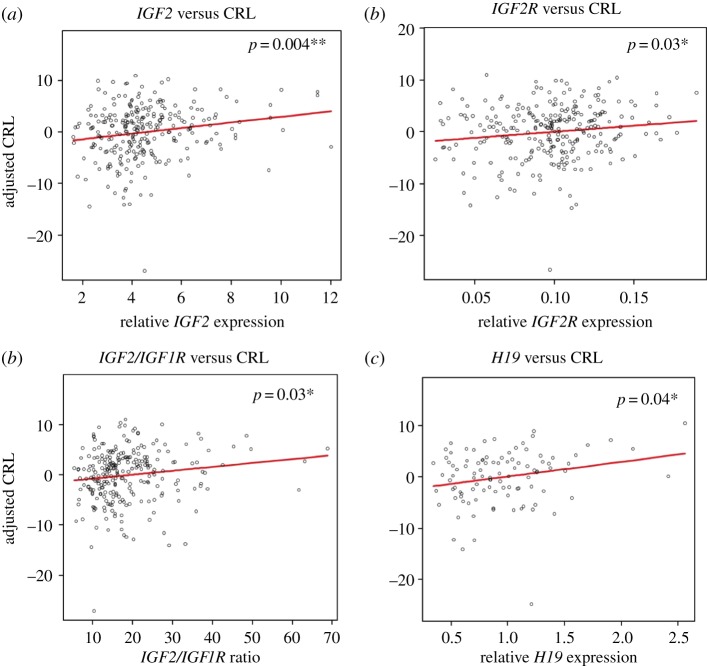
Correlation between imprinted gene expression in CVS and CRL. Expression levels of each gene relative to the *L19* endogenous control gene were correlated to crown–rump length (CRL: mm) using a multiple linear regression model adjusted for maternal BMI, baby's sex, parity, gestational age when CRL was measured and maternal smoking habit. Positive correlations with CRL and (*a*) *IGF2* expression (*r* = 0.77; *p* = 0.004), (*b*) *IGF2R* expression (*r* = 0.76; *p* = 0.03), (*c*) *IGF2/IGF1R* ratio (*r* = 0.74; *p* = 0.03) and (*d*) *H19* expression (*r* = 0.74; *p* = 0.04) were observed. (Online version in colour.)

In our previous studies, we have shown that *IGF2* and *IGF2R* expression in term placenta has no correlation with baby's birth size parameters. However, their expression levels in CVS tissues showed a strong positive correlation with birthweight [17,33], indicating their role as ‘early growth effectors'. In addition to this study, the expression levels of *H19* (*n* = 104) relative to the ribosomal protein L19 (*L19*) endogenous control gene in CVS tissues was measured by RT-quantitative polymerase chain reaction (qPCR). The relative expression levels of *H19* were correlated to birth weight in a regression model adjusted for baby's sex, parity, gestational age at birth, maternal BMI and smoking habits. The CVS expression data for *IGF2*, *IGF2R*, *H19*, *PHLDA2*, *IGF1* and *IGF1R* were also correlated to CRL at the gestational age of 12 weeks, using the same regression model, except this time the gestational age at CRL measurement was used instead of gestational age at birth. Correlation between *H19* expression and birthweight was not statistically significant (*p* = 0.07). However, there was significant evidence for positive association between CRL at 12 weeks and *IGF2* expression (*p* = 0.004; figure 1*a*), *IGF2R* expression (*p* = 0.03; figure 1*b*), *IGF2*/*IGF1R* ratio (*p* = 0.03; figure 1*c*) and *H19* expression (*p* = 0.04; figure 1*d* and table 3). These results suggest that the many members of the IGF axis (*IGF2*, *IGF2R* and *IGF1R*), and the closely associated *H19*, shape the growth trajectory early in pregnancy.

**Table 3.** The association between mRNA levels and fetal growth in term placenta and CVS. Shading indicates previously published results [17,33]. The correlation significance is indicated by *p*-values. Correlation coefficient (*r*) is presented underneath the *p*-values for the associations reaching significance. *n*, number of samples; BW, birth weight; PW, placental weight; HC, head circumference; CRL, crown–rump length; NT, not tested.

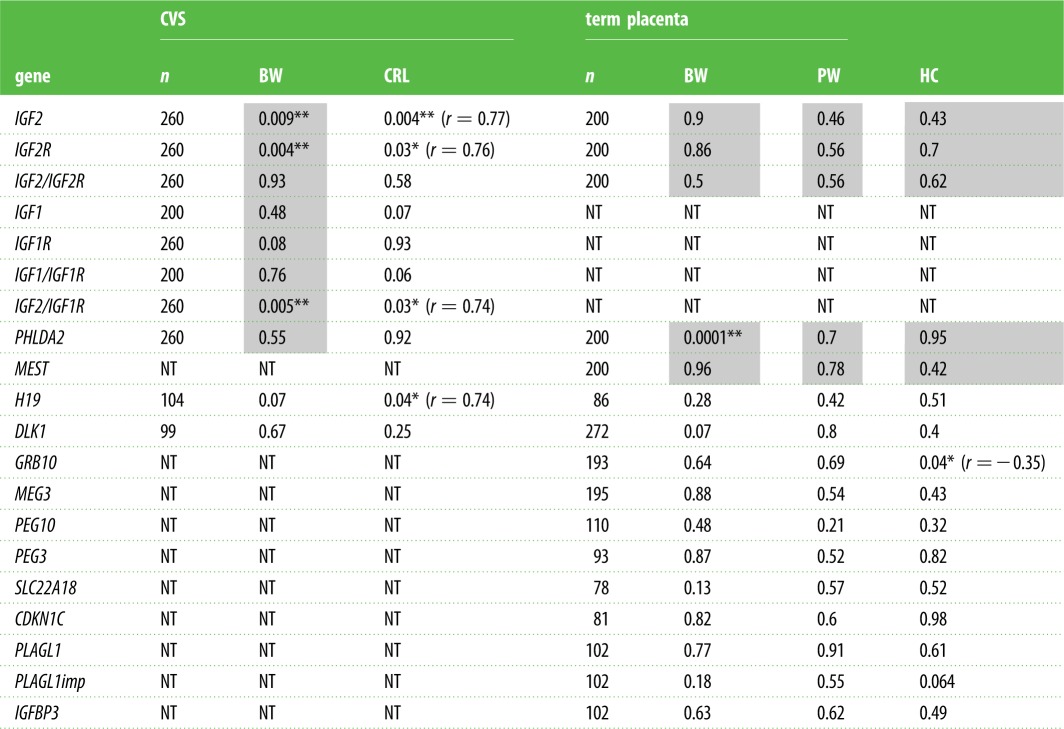

There was no correlation between maternal smoking and the expression in CVS of the genes tested (those listed above) in our samples. Nevertheless, we observed an association between *IGF2* expression and parity, whereby *IGF2* expression is higher in the ‘parity greater than one’ group of babies (*p* = 0.03; electronic supplementary material, figure S1*a*); this is consistent with the role of *IGF2* as a positive growth regulator. This observation is interesting as the majority of second born babies are bigger [36]. We also found evidence that the maternal BMI was positively correlated with *IGF2R* expression (*p* = 0.03; electronic supplementary material, figure S1*b*) and negatively correlated with *IGF1* (*p* = 0.046; electronic supplementary material, figure S1*c*). This suggests that it is important to allow for correction for maternal BMI/weight when investigating the gene expression in association with fetal growth. Interestingly, *H19* was expressed significantly higher in males (*p* = 0.006; electronic supplementary material, figure S1*e* and table S2). The observed sex bias cannot be explained by LOI (i.e. biallelic expression of *H19* in males only), because all of the CVS tissues tested retained monoallelic expression (table 4), therefore it is likely to result from upregulation of the active maternal copy. Males are normally born bigger than females [36], and the sexual dimorphism in antenatal biometry has been reported to be evident around 8–12 weeks of gestation [53]. As *H19* is a negative growth regulator, the higher expression may help prevent male babies from growing too large.

**Table 4.** Summary of imprinting analysis in CVS tissues and term placenta. M, maternal expression; P, paternal expression. %, percentage of samples with monoallelic expression within informative samples; n.a., not available.

gene	parental origin	imprinting in term placenta	imprinting in CVS	polymorphic site
*IGF2*	P	67/67 (100%)	40/40 (100%)	rs680
*IGF2R*	M/biallelic	n.a.	3/24 (12%)	rs1805075
*PHLDA2*	M	11/11 (100%)	21/21 (100%)	rs13390, rs1056819
*MEST*	P/biallelic	34/42 (81%)	n.a.	rs10863
*H19*	M	19/19 (100%)	33/33 (100%)	rs2067051
*DLK1*	P	30/30 (100%)	n.a.	rs1802710
*MEG3*	M	9/9 (100%)	n.a.	rs45617834, rs941575
*PEG3*	P	14/16 (88%)	n.a.	rs1055359
*PEG10*	P	42/42 (100%)	n.a.	rs13073, rs13226637
*GRB10*	M (placenta), P (brain)	n.a.	n.a.	n.a.
*SLC22A18*	M	23/23 (100%)	n.a.	rs1048046, rs1048047
*PLAGL1*	P	11/11 (100%)	n.a.	rs2076684
*CDKN1C*	M	24/24 (100%)	n.a.	PAPn repeat

### (d) GBR10

*GRB10* is located in the human chromosome 7q12 imprinted region. Chromosome 7 is implicated in causality for SRS, because 10% of patients show maternal uniparental disomy of chromosome 7. FGR is a key feature of SRS, which has been suggested to result either from the overexpression of a maternally expressed gene or loss of a paternally expressed growth-promoting gene. *GRB10* encodes a growth factor receptor binding protein that can interact with receptor tyrosine kinases and intracellular proteins [54]. *GRB10* is imprinted in an isoform- and a tissue-specific manner [55]. In humans, *GRB10* shows biallelic expression in most tissues, while exhibiting isoform-specific paternal expression in the brain but with maternal expression confined to the placental villous trophoblast [56]. In mice, *Grb10* is paternally expressed in the brain, but shows ubiquitous maternal expression in other tissues [55]. This pattern is roughly the opposite of what is seen for *Igf2,* where it is preferentially maternally expressed in the adult mouse brain but paternally expressed in other tissues [57]. Inactivation of the maternal copy of *Grb10* results in fetal and placental overgrowth, indicative of its role as a potent growth suppressor [10]. In contrast, mice with a disrupted paternal copy showed normal growth but increased social dominance behaviour, illustrated by increased facial barbering (whisker removal) on cage-mates [58].

**Figure 2.**
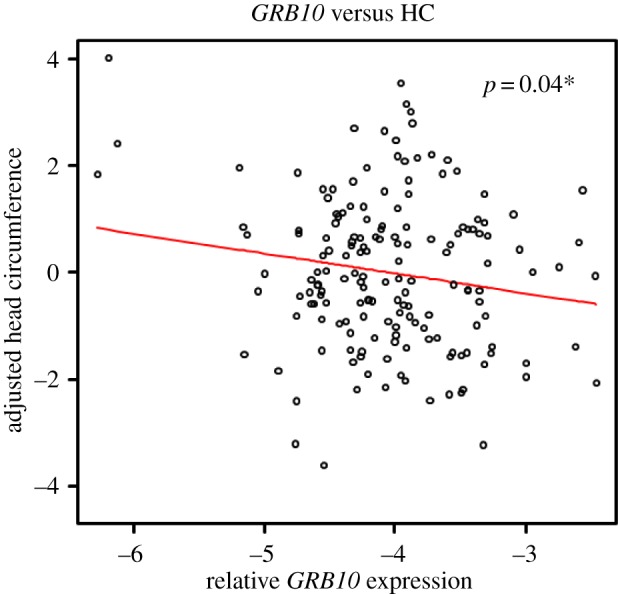
Negative correlation between *GRB10* term placental expression and head circumference. The expression level of *GRB10* relative to the *L19* housekeeping gene was correlated to head circumference (cm) using a multiple linear regression model adjusted for baby's sex, parity, gestational age at birth, maternal weight and smoking habits. *GRB10* expression values in logarithmic scale was used. Significant negative association was observed for *GRB10* term placenta expression and head circumference (*r* =−0.35; *p* = 0.04). (Online version in colour.)

In this study, we have observed a significant negative association between *GRB10* expression (all isoforms) and head circumference (figure 2, *p* = 0.04), but no significant correlation with birthweight (*p* = 0.64) or placental weight (*p* = 0.69; table 3). The direction of association is consistent with the role of *GRB10* as a negative growth regulator. It is interesting that the observed association is specific to head circumference, because it is oppositely imprinted in the brain. There was no correlation between maternal smoking and expression of the genes tested in term placenta samples (electronic supplementary material, table S3). Interestingly, *GRB10* expression showed a positive association with increasing gestational age (*p* = 0.03; electronic supplementary material, figure S2*a*). This suggests that *GRB10* is acting to suppress the head circumference of the baby close to birth, because a head size too large for the birth canal would be detrimental for the mother.

### (e) PHLDA2

*PHLDA2* is a maternally expressed gene located on the centromeric domain of the Chr11p15 imprinting cluster, along with other maternally expressed genes *CDKN1C* and *SLC22A18. PHLDA2* encodes a small (144 amino acid) protein with a Pleckstrin-homology (PH) domain which has the capacity to bind membrane phosphatidylinositol phosphate lipids (PIPs) [59], suggesting a role for it as a cell signalling protein. In line with the kinship theory, *Phlda2*-deficient mice have an enlarged placenta, whereas overexpression of *Phlda2* in transgenic mice results in placental stunting with a modest reduction in fetal weight [60,61]. We have previously shown that birth weight is not correlated with *PHLDA2* expression levels in CVS tissues, but has a significant negative correlation in term placenta [17,33], indicative of a function as a ‘late growth effector’. Other studies have observed upregulation of *PHLDA2* in FGR placentas [21,34,35], and in first and second trimester miscarriage placentas [62]; these data all support the hypothesis that *PHLDA2* is an important negative regulator of growth.

More recently, upregulation of placental *PHLDA2* expression among mothers who smoke during pregnancy has been reported [63]. In our study, however, we did not observe any correlation between maternal smoking and CVS or term placental expression of *PHLDA2* (electronic supplementary material, table S3). *PHLDA2* expression in CVS and term placenta did not show correlation with any of the confounding variables used in the model, except for gestational age. We identified that reduced *PHLDA2* expression in CVS tissues was associated with advancing gestational age at birth (*p* = 0.0092; electronic supplementary material, figure S1*e*). Because a shorter gestation results in smaller babies, its high expression in CVS fits its role as a growth suppressor.

All the samples used in the analysis showed monoallelic expression of *PHLDA2*, demonstrating that LOI cannot account for the increased expression seen in the smaller birth weight babies [33]. To further investigate this correlation, we successively interrogated the nearby region for potential genetic variations that correlate with fetal growth. We identified a rare 15 bp repeat sequence variant (RS1) in the *PHLDA2* promoter region, which has been shown to reduce the *PHLDA2* promoter efficiency [36]. Maternal inheritance of RS1 resulted in a 93 g increase in birthweight, and when the mother is homozygous for RS1, the effect on birthweight is 155 g, suggesting a grand-maternal influence. Paternal inheritance of RS1 does not influence fetal growth as the variant lies on the epigenetically silenced paternal allele, emphasizing the importance of taking into account parent-of-origin effects when analysing genetic variants. Taken together, these data show that *PHLDA2* is a strong negative growth suppressor and provide a potential pre-pregnancy test, using the RS1 variant, to predict birthweight.

### (f) DLK1

*DLK1* (*PREF1* and *FA1*) is a paternally expressed gene located in the human chromosome 14q32 imprinting cluster, approximately 90 kb away from the maternally expressed non-coding RNA gene *MEG3* (also called *GTL2*). *DLK1* encodes a transmembrane glycoprotein with six epidermal growth factor-like repeat motifs [64], known to be involved in adipogenesis [65]. *Dlk1*-null mice show high perinatal lethality, pre- and postnatal growth restriction followed by an obese phenotype [37], suggesting that it acts as a growth promoter.

In this study, the expression levels of *DLK1* (all isoforms) in CVS (*n* = 99) and term placenta (*n* = 272) were correlated to fetal growth parameters. For the CVS analysis, only the tissues from extreme birthweight babies (less than 10th centile and more than 90th centile) were used. Using the regression model as described for *H19*, we did not observe any association between *DLK1* expression and birthweight (*p* = 0.23) or with CRL (*p* = 0.16). However, term placental *DLK1* expression did show a weak positive association with birthweight (*p* = 0.07; table 3). Although this trend did not reach statistical significance, the direction of influence is consistent with its role as a growth promoter. Interestingly, *DLK1* expression showed a positive correlation with increasing parity (*p* = 0.05; electronic supplementary material, figure S2*b* and table S3), possibly increasing the size of the later parity babies.

The rs941576 (G/A) SNP of the *DLK1*-*MEG3* gene region on human chromosome 14 has previously been identified as a type 1 diabetes (T1D) susceptibility locus [38]. A reduced paternal, but not maternal, transmission of the protective G allele was observed in the T1D-affected individuals, showing a clear parent-of-origin effect. It was suggested that the rs941576 variant may affect nearby paternally expressed genes, including *DLK1*. Notably, higher birthweight has been linked to increased T1D risk [66–68]. This prompted us to test whether paternal transmission of the protective G allele is associated with (i) lower *DLK1* expression and/or with (ii) reduced birthweight using the DNA samples from the Moore cohort. Because this is located within intron 6 of *MEG3* and 105 kb downstream of *DLK1*, its potential influence on *MEG3* expression was also tested.

In this study, 295 trio DNA samples from the Moore cohort were used for genotyping the rs941576 SNP. The resulting frequencies of the three genotypes were GG: 24%, AG: 45% and AA: 31%. 112 and 141 babies inherited paternal G and A bases, respectively, and 119 and 132 babies inherited maternal G and A, respectively. Using multiple linear regression analysis, we found that paternal or maternal transmission of the G allele is not correlated with *DLK1* expression (*p* = 0.47 and *p* = 0.63, respectively) or with *MEG3* expression (*p* = 0.7 and *p* = 0.085, respectively).

**Figure 3.**
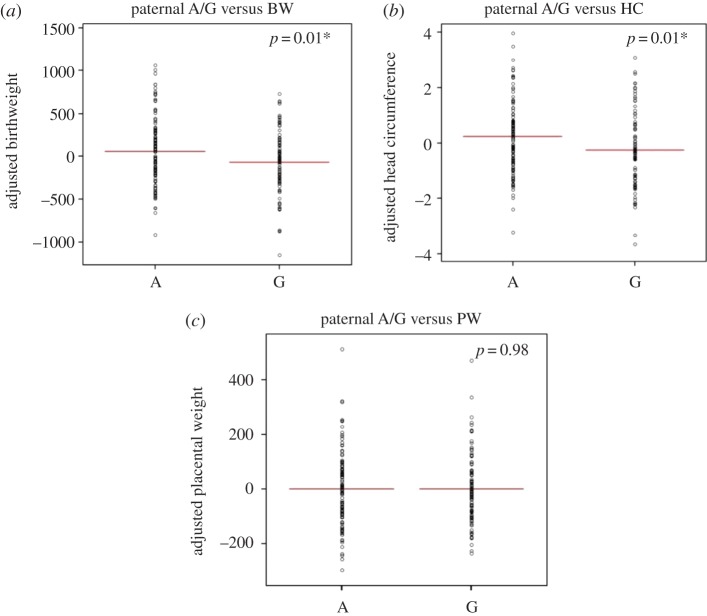
The association between paternal A/G SNP rs941576 at the *DLK1* locus and fetal growth. Partial residual plots illustrating the correlation between paternal inheritance of the A or G allele and (*a*) birthweight (g), (*b*) head circumference (cm) and (*c*) placental weight (g), corrected for baby's sex, parity, gestational age, maternal weight and smoking habit in the multiple regression model. In comparison to the A allele, paternal G allele inheritance is associated with significantly reduced birthweight (*p* = 0.01, 95% CI−232 to −32) and head circumference (*p* = 0.01, 95% CI −0.85 to −0.11) but not with placental weight (*p* = 0.98, 95% CI −35 to 35). Paternal A/G, paternal transmission of A/G SNP rs941576; A, paternal transmission of the A allele; G, paternal transmission of the G allele; BW, birthweight; HC, head circumference; PW, placental weight. (Online version in colour.)

Next, the association between the inheritance of a paternal G allele with fetal growth was investigated, using a multiple linear regression model, adjusted for sex of the baby, parity, gestational age and maternal weight and smoking habit. Paternal transmission of the G allele was significantly associated with an average decrease of birthweight by 132 g (*p* = 0.01, 95% CI− 232 to −32; figure 3*a*), and a 0.5 cm reduction in head circumference of the baby (*p* = 0.01, 95% CI −0.85 to −0.11; figure 3*b*), but not with placental weight (−0.45 g; *p* = 0.98, 95% CI −35 to 35; figure 3*c*). Importantly, the scale of birthweight reduction (−132 g) associated with paternal G transmission is similar to that of the maternal smoking (−152 g). Maternal inheritance of the G or A allele was not associated with birthweight (*p* = 0.8), head circumference (*p* = 0.62) or placental weight (*p* = 0.86), consistent with the observed paternal effect of the protective G allele in T1D susceptibility.

### (g) Other imprinted genes studied

No evidence of correlation between *H19*, *MEG3*, *PEG10*, *PEG3*, *SLC22A18*, *CDKN1C*, *PLAGL1*_imp (imprinted transcript), *PLAGL1*_all (all transcripts) or *IGFBP3* expression, in term placenta, with fetal growth was observed (summarized in table 3). In addition, we were unable to corroborate a previously reported association between *SLC22A18* expression and head circumference [31]. We did not observe any LOI in our samples, except for *PEG3*, where 2/16 (12%) samples showed biallelic expression in term placenta. Table 4 details the polymorphic variants used for each gene and imprinting analysis results.

It was not possible to test all the candidate genes in both term placenta and for CVS tissues, owing to the limited availability of material for the latter, whereas some candidates also showed a level of expression undetected by quantitative PCR. Therefore, the candidate genes have been prioritized according to their functional relevance. Although it would have been interesting to test *GRB10* expression in CVS, head circumference measurements were not available for the KCL CVS cohort.

## 2. Discussion

Suboptimal or excessive intrauterine growth leads to perinatal morbidity and mortality, as well as an increased risk for adulthood diseases [4]. Finding genetic factors that regulate normal fetal growth will potentially provide more precise monitoring of intrauterine growth. Genomic imprinting epigenetically silences one parental allele resulting in monoallelic expression. It is now accepted that paternally expressed genes tend to encourage fetal growth, whereas maternally expressed genes restrict this. In this paper, the role of imprinted genes on fetal growth was explored by summarizing and connecting our previous and current findings. Although DNA methylation plays a key regulatory role in imprinted gene expression, their methylation statuses were not assessed in our samples as CVS is a limited resource. RNA expression variation is downstream of DNA methylation or other possible DNA regulatory factors, and therefore potentially more functionally relevant. An additional DNA methylation status assessment would be an interesting aspect for the future study.

### (a) The early and late effectors of fetal growth

Combining past and present studies, we have investigated the correlation between fetal growth measurements and expression levels of 13 imprinted and three non-imprinted genes highly expressed in CVS tissues and term placenta (tables 1 and 2). Our candidate gene approach has identified some early and late effectors of fetal growth. We have shown that the CVS expression of *IGF2* and *IGF2R* is positively correlated to birthweight, whereas this correlation disappears in term placenta (table 3). Conversely, *PHLDA2* expression in CVS is not correlated to birthweight, whereas *PHLDA2* expression at term is strongly negatively correlated to birthweight. Although *GRB10* expression in CVS was not tested, its expression in term placenta showed a strong negative association with head circumference. These observations suggest that *IGF2* and *IGF2R* can act to set the growth potential of the baby early in the pregnancy, and two maternally expressed growth suppressing genes, *PHLDA2* and *GRB10,* act to fine tune growth in late pregnancy, potentially to avoid the risk of giving birth to a macrosomic baby. Importantly, mouse studies indicate that both *Phlda2* and *Grb10* control placental growth by mechanisms independent of *Igf2* [10,32], implying the evolution of separate pathways to control overall fetal size, possibly reflected by the difference in timing of their functional action.

The first half of placental development is characterized by a series of important trophoblast proliferation and differentiation processes, forming mature villous and extravillous structures. The second half of gestation results in an extensive vascularization and placental mass expansion [69]. Early gestational insults such as maternal diabetes have been associated with long-term effects on the fetus, owing to their influence on the initial structural formation of the placenta. It is possible that *IGF2* and *IGF2R* are key regulators of early formation of the placenta, which then sets the growth capacity of the fetus and placenta for the rest of gestation. Interestingly, overexpression of mouse *Phlda2* results in placental size reduction, with decreased glycogen storage and failed mobilization, accompanied by progressive fetal weight loss in late gestation [61]. It has been suggested that halving *Phlda2* expression by silencing the paternal allele later in gestation may promote energy provision for the fetus at this time, by increasing the glycogen stores that will be used in late gestation when there is a particularly high nutrient demand from the fetus [61].

### (b) Environment and other physiological effectors on gene expression

Although placenta is fetal in origin, it is under the influence of both maternal and fetal circulation. The placental villi consist of syncytiotrophoblasts facing the maternal blood, with cytotrophoblasts in the middle and endothelial cells facing the fetal circulation [69]. Therefore, the mRNA measured in the placenta could be a result of response to the hormones and growth factors present in both maternal and fetal circulation. In this study, potential influences of environmental variations (maternal weight/BMI and maternal smoking) and physiological variation (baby's gender, gestational age and parity) on gene expression were tested.

We did not observe a correlation between maternal smoking and gene expression levels with all genes tested in both CVS and term placenta (electronic supplementary material, table S2). This result contradicts the previous report where the upregulation of placental *PHLDA2* in smokers (*n* = 12) compared with non-smokers (*n* = 64) was observed in a microarray experiment [63]. This could be due to different sensitivities between the two techniques. However, our cohorts contained more smokers (*n* = 27, Moore cohort and *n* = 33, CVS cohort; electronic supplementary material, table S1), which allows for more accurate measure of expression. *IGF2R* expression in CVS showed a positive association with maternal BMI (electronic supplementary material, figure S1*b* and table S2). This is interesting, because *IGF2R* has been found in the syncytiotrophoblast, which is in direct contact with the maternal blood circulation, and therefore possibly regulating the effect of fetal *IGF2* levels on the mother [70].

Notably, we have found a sex-biased expression of *H19* in CVS tissues, where it is expressed more highly in males (electronic supplementary material, figure S1*d*). *H19* has previously been reported to show female-biased expression in mouse eyes [71]. Therefore, *H19* expression could be dually regulated according to the sexes of the parent (imprinting) and also the baby (sexual dimorphism), in a tissue- and time-specific manner. Moreover, downregulation of *PLAGL1* in FGR placenta of females, but not males, has been reported [72]. This was not evident in our normal term placenta samples, implying FGR-specific effects. Insight into the effect of sexual dimorphism is important for understanding both normal molecular mechanisms and sex-biased disease conditions.

### (c) *DLK1*, type 1 diabetes and parent-of-origin effect on fetal growth

Type 1 diabetes (T1D) is caused by autoimmune destruction of pancreatic beta cells, resulting in insulin deficiency, although its aetiology is not fully understood [68]. The *DLK1*-*MEG3* imprinting locus has recently been identified as a T1D susceptibility region, marked by the rs941576 SNP in which paternal inheritance of a G allele was associated with reduced risk [38]. *DLK1* is highly expressed in pancreatic islet cells and is involved in differentiation of pancreatic beta cells, suggesting its strong functional candidacy [73].

**Figure 4.**
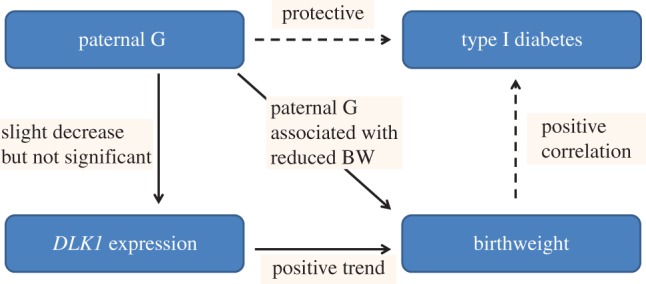
Current hypothesis on the association between paternal G SNP rs941576 and fetal growth. Solid lines indicate results from this study and dotted lines indicate published data [41–43]. Paternal inheritance of the G allele is associated with an average reduction in birthweight by 132 g (*p* = 0.01). The paternal G allele is also correlated with reduction in *DLK1* expression although not significantly (*p* = 0.47). There was a trend of positive association between *DLK1* expression in term placenta and birthweight (*p* = 0.07). Our hypothesis suggests that the paternal G allele reduces *DLK1* expression which causes reduction in birthweight and risk of type 1 diabetes. (Online version in colour.)

In this study, we found that paternal transmission of the protective G allele results in a significant decrease of birthweight, by 132 g (figure 3*a*), and head circumference, by 0.5 cm (figure 3*b*). Of note, higher birthweight has been linked to increased T1D risk [66–68]. Therefore, paternal inheritance of the G allele may give protective effect from T1D via its association with reduced birthweight. This could also be associated with a decrease in *DLK1* expression although this association did not reach statistical significance. Importantly, the magnitude of birthweight reduction (−132 g) and head circumference (−0.5 cm) related to the paternal G allele inheritance was similar to that observed for the increase in birthweight (+155 g) and in head circumference (+0.23 cm) caused by inheriting a *PHLDA2* promoter RS1 allele from a RS1 homozygous mother [36]. Our current working hypothesis regarding the relationship between the role of *DLK1* in fetal growth and T1D is described in figure 4 [37,64,65].

## 3. Conclusion

We have identified that expression of *IGF2* and *IGF2R* in early placenta (CVS) are positively correlated to CRL and birthweight, but not in term placenta when the oppositely maternally expressed genes *PHLDA2* and *GRB10* act to negatively regulate growth. We have also identified that the paternal transmission of the T1D protective G allele of rs941576 SNP results in a significant reduction in birthweight (*p* = 0.01, 95% CI−232 to −32), emphasizing the importance of accounting for parent-of-origin effects when analysing genomic data. Characterization of genes important in intrauterine growth will allow a more accurate surveillance of fetal growth and help identify targets for clinical intervention in suboptimal pregnancies. During pregnancy, a combination of different levels of imprinted genes or genetic predispositions will affect the baby's birthweight. An additional environmental layer is added by maternal smoking. Further investigation of all these candidates is warranted in larger cohorts to identify further genetic variants that exhibit parent-of-origin associated growth regulation and to find gene expression variations. Together with previously known genetic variants associated with fetal growth (reviewed in [26]), and expression studies, these may be used as an effective, combined diagnostic tool to identify and predict growth-restricted and macrosomic babies, which would provide huge benefits for the short- and long-term health of both mother and baby.

## 4. Materials

### (a) King's College London chorionic villus sample cohort

CVS was carried out between 11 and 13 weeks of gestation in 355 singleton pregnancies that were followed by normal live birth at term. Participants were undergoing CVS for prenatal diagnosis for chromosomal abnormality at King's College Hospital London. The samples used in this study were obtained from excess CVS tissues from fully ethically consented women, and the research was approved by the King's College Hospital Ethics Committee. The medical records of this cohort are summarized in the electronic supplementary material, table S1 [17].

### (b) Moore cohort

The Moore cohort consists of 302 consented white European trios recruited at Queen Charlotte's and Chelsea Hospital between 2003 and 2004 [33]. The placental samples were collected from ultrasound dated, live birth singleton pregnancies. Each placental sample was dissected into four pieces near the umbilical cord insertion point, washed in phosphate-buffered saline, snap-frozen in liquid nitrogen and stored at −80°C. Parental blood samples (10 ml) were collected in EDTA tubes. The medical records and characteristics of the Moore cohort are summarized in the electronic supplementary, table S1.

## 5. Methods

### (a) DNA and RNA extraction

Total RNA from term placental tissue was extracted using Trizol reagent (Life Technologies), and treated with TURBO DNase (Ambion) according to the manufacturers guidelines. Fetal DNA from 1 g of term placental tissue and parental DNA from 2 ml of whole blood were isolated using a standard phenol–chloroform protocol. RNA and DNA from CVS tissues were extracted by the iPrep PureLink total RNA and TrizolPlus RNA kit, including the DNase treatment and iPrep ChargeSwitch gDNA tissue kit using the iPrep purification instrument (Life Technologies) following the manufacturer's instructions. The quantity and purity of nucleic acid was measured by NanoDrop ND-1000 spectrophotometer (Thermo Scientific). Only RNA samples with the 260/260 ratio in the range of 2 ± 0.2 were used for further study.

### (b) Reverse transcription

A first strand of complementary DNA (cDNA) was synthesized from 1 μg (term placenta) and 100 ng (CVS) of RNA with Moloney murine leukemia virus reverse transcriptase (M-MLV RT) according to the manufacturer's instructions (Promega). Duplicate sets of samples without reverse transcriptase were made as negative controls to detect any genomic contamination in RNA samples. The conversion of RNA to cDNA was confirmed by polymerase chain reaction (PCR) with Taq DNA polymerase (Bioline) beta-actin (*ACTB*) primers (electronic supplementary material table S5).

### (c) Quantitative polymerase chain reaction

qPCR was performed using the *Power* SYBRGreen PCR master mix (Life Technologies). Each sample was tested in triplicate, and each plate contained a no-template-control and a cDNA pool as a reference sample to control for interplate variations. The reaction plate was placed on the StepOne plus real-time PCR systems, analysed in the comparative *C*_t_ mode. Ribosomal protein L19 (*L19*) housekeeping gene was used as an endogenous control throughout the experiments. Thermal cycle conditions consist of initial incubation at 50°C for 2 min for one cycle, polymerase activation at 95°C for 10 min for one cycle and 40 cycles of denaturation at 95°C for 15 s, and annealing and extension at 60°C for 1 min. The efficiency of the primers was determined by running a standard curve and calculated by 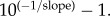 The qPCR primer sequences are provided in the electronic supplementary material, table S4. The resulting data were analysed with the StepOne v. 2.1 software to obtain relative quantification (RQ) values, using the formula RQ **=** 2^−ΔΔ*C*t^.

### (d) Imprinting analysis

Monoallelic expression of genes was investigated by sequencing gene-specific amplicons from cDNA samples that corresponded to genomic DNA heterozygous for selected SNPs. Parental DNA was available for term placental samples, and was used for sequencing to check the parental origin of the expressed allele. SNPs with relatively high average heterozygosity were chosen for each gene within the exon covering all isoforms. PCR primer sequences are summarized in the electronic supplementary material, table S5, and the list of selected SNPs is found in table 4. Sequencing was carried out using the BigDye terminator v. 1.1 cycle sequencing kit (Life Technologies), and the read-out was analysed with Sequencher v. 4.8 (Gene Codes Corporation).

### (e) Statistical analysis

All statistical analyses were performed using the R software (R Foundation for Statistical Computing). The relative expression of the candidate genes in term placenta was correlated to the baby's birth weight, placental weight and head circumference using a multiple linear regression model adjusted for baby's sex, gestational age, parity, maternal weight/BMI and smoking habits. These variables used in the model have previously been established as confounding factors in our previous studies in the same cohort [33,36]. A logarithmic scale was used for the expression values when appropriate, and BIC test was performed to check the fit of the models. A significance threshold of 5% was used in the analysis.

## Supplementary Material

Supplementary data
