# Supplementary material for: The role and interaction of imprinted genes in human fetal growth
Source: Philos Trans R Soc Lond B Biol Sci. 2015 Mar 5;370(1663):20140074. doi: 10.1098/rstb.2014.0074 (PMC4305174; doi:10.1098/rstb.2014.0074)
Supplement: Supplementary data [file rstb20140074supp1.pdf]

Electronic supplementary material

The Role and Interaction of Imprinted Genes in Human Fetal Growth

Gudrun E. Moore, Miho Ishida, Charalambos Demetriou, Lara Al-Olabi, Lydia J. Leon, Anna C. Thomas, Sayeda Abu-Amero, Jennifer M. Frost, Jaime L. Stafford, Yao Chaoqun, Andy Duncan, Rachel Baigel, Marina Brimioulle, Isabel Iglesias-Platas, Sophia Apostolidou, Reena Aggarwal, John C. Whittaker, Argyro Syngelaki, Kypros H. Nicolaides, Lesley Regan, David Monk, Philip Stanier

Journal: Philosophical Transactions B
DOI: 10.1098/rstb.2014.0074

Contents:

Figure S1: Association between CVS gene expression and factors influencing fetal growth ………………..2

Figure S2: Association between term placenta gene expression and factors influencing fetal growth……….3

Table S1: Medical details of participants in the Moore and the KCL CVS cohorts…………………………..4

Table S2: Correlation between gene expression in CVS and variables used in the regression model………...5

Table S3: Correlation between gene expression in term placenta and variables used in the regression model.5

Table S4: Primer sequences for RT-qPCR……………………………………………………………………6

Table S5: Primer sequences for the imprinting analysis……………………………………………………...7

References ……………………………………………………………………………………………………7

Supplementary figures

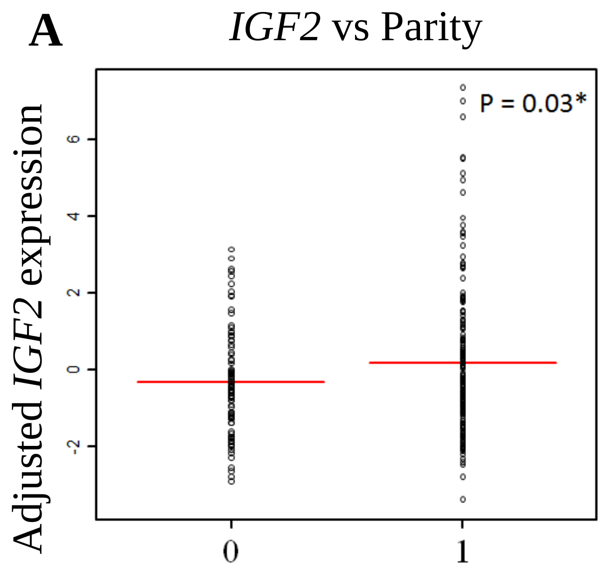

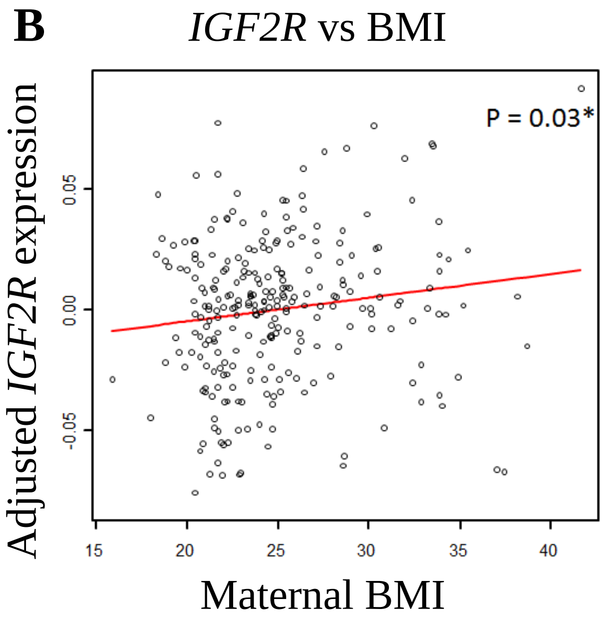

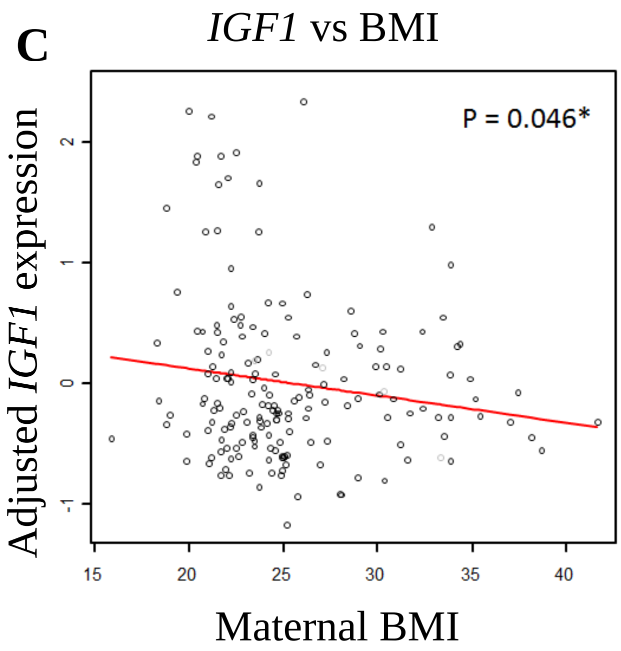

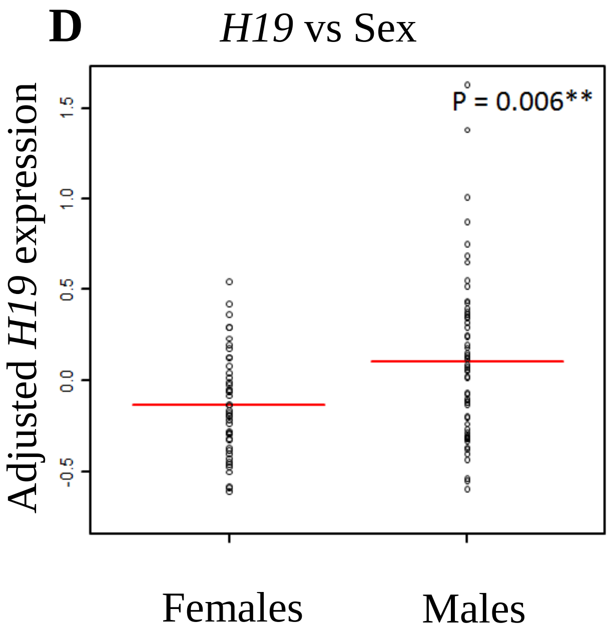

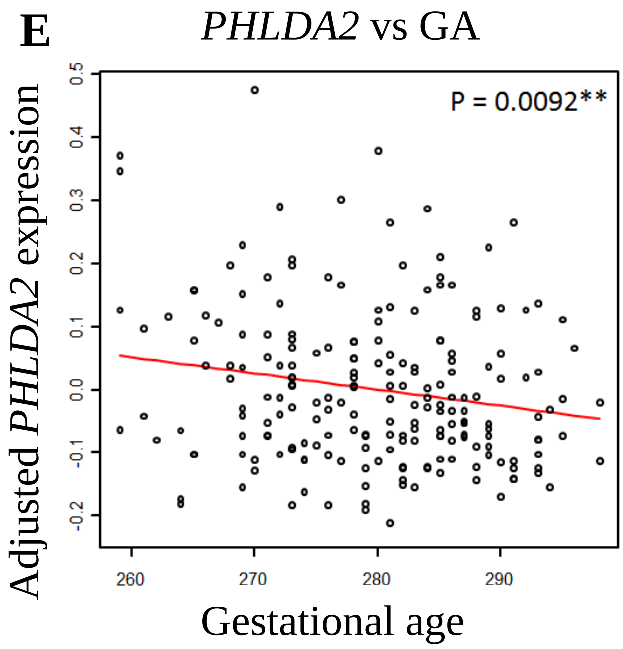

Supplementary Figure 1. Association between CVS gene expression and factors influencing fetal growth. The associations between gene expression in CVS tissue and the following variables were tested in the regression model; baby's sex, parity, gestational age at birth, maternal BMI and smoking. (A) *IGF2* is expressed higher in the parity > 1 group (P = 0.03). Maternal BMI is positively and negatively correlated to (B) *IGF2R* (P = 0.03) and (C) *IGF1* (P = 0.046). (D) *H19* is expressed higher in males (P = 0.006). (E) *PHLDA2* expression is higher as gestational age at birth (in days) advances (P = 0.0092).

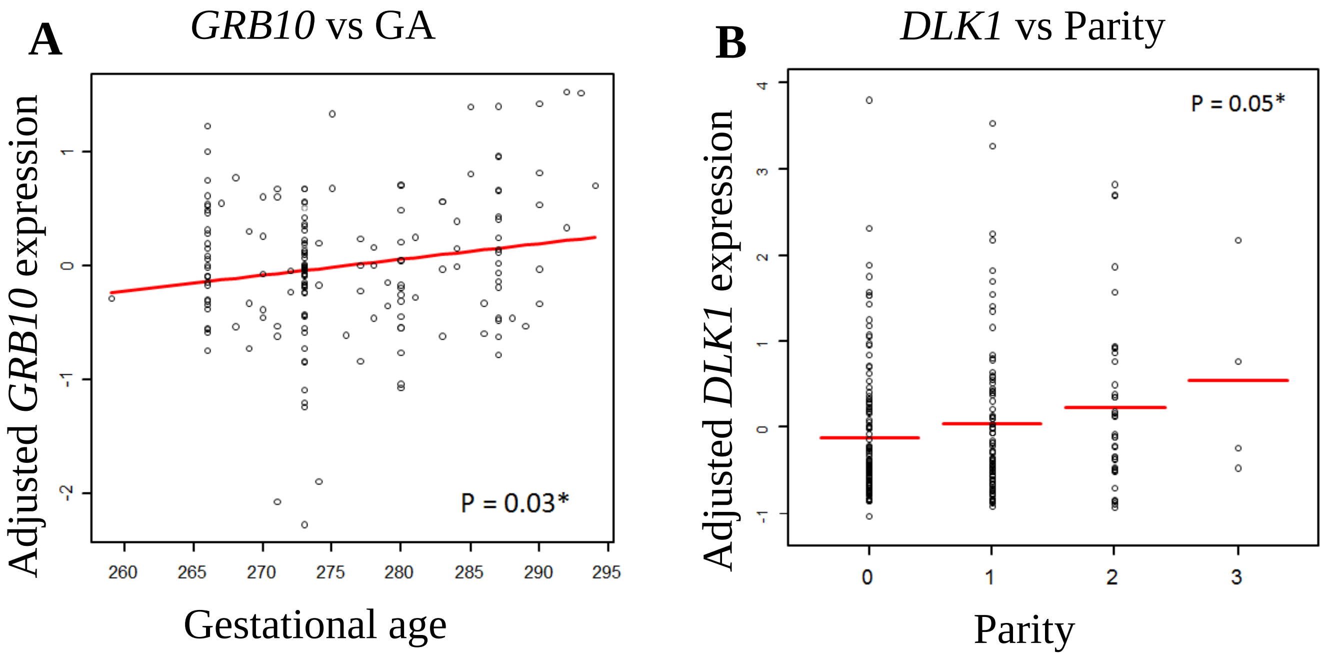

Supplementary Figure 2. Association between term placenta gene expression and factors influencing fetal growth. The influence of baby's sex, parity, gestational age at birth, maternal weight and smoking on imprinted genes expression in term placenta was tested using a regression model. Partial residual plots showing a positive correlation between gestational age (in days) at birth and (A) *GRB10* expression (P = 0.03). Parity is positively correlated to (B) *DLK1* expression (para 1 P = 0.47, para 2 P = 0.05 and para 3 P = 0.12).

Supplementary tables

Supplementary Table 1. Medical details of participants in the Moore and the KCL CVS cohorts.

Cohorts	Moore cohort	KCL CVS cohort
Sample number	302 trios	355
Maternal information:		
Pre-pregnancy weight (Kg)	67 ± 11.7	67 ± 13
Maternal BMI	NA	25 ± 4.4
Maternal age (years)	34 ± 5	35.7 ± 5.6
Smoking (% smokers)	27 (8.9 %)	33 (9 %)
Parity	0: 148 (49 %)	0: 93 (26 %)
	1: 99 (32 %)	>1: 177 (50 %)
	>2: 55 (18 %)	NA: 85 (24 %)
Baby's information:		
Ethnicity:		
White European	302 (100 %)	251 (71 %)
Black		19 (5%)
Asian		11 (3%)
Oriental		9 (3%)
Unknown		65 (18 %)
Gestational age at birth	39 ± 1 (weeks)	279.4 ± 9 (days)
Gestational age at CVS	NA	89.6 ± 4.2
Gender (% males)	51 %	54 %
Crown rump length (mm)	NA	66.9 ± 8
Birth weight (g)	3,432 ±453	3,507 ± 636
Placental weight (g)	702 ± 137	NA
Head circumference (cm)	34.9 ± 1.5	NA

Mean ± standard deviations. NA = information not available.

Supplementary Table 2. Correlation between gene expression in CVS and variables used in the regression model.

Gene	Mat BMI	Sex	Parity	GA at term	Smoking
IGF2	0.45	0.38	**0.03***	0.39	0.42
IGF2R	**0.03***	0.62	0.56	0.33	0.48
IGF1	**0.046***	0.13	0.07	0.89	0.99
IGF1R	0.067	0.97	0.87	0.5	0.45
PHLDA2	0.92	0.62	0.63	**0.0092****	0.28
DLK1	0.74	0.28	0.71	0.11	0.88
H19	0.14	**0.006***	0.95	0.65	0.74

GA = gestational age (days), BMI = body mass index.

Supplementary Table 3. Correlation between gene expression in term placenta and variables used in the regression model.

Gene	MW	Sex	Para 1	Para 2	Para 3	GA	Smoking
IGF2	0.31	0.42	0.12	0.098	0.85	0.54	0.29
IGF2R	0.88	0.56	0.2	0.71	0.43	0.53	0.41
PHLDA2	0.099	0.084	0.12	0.83	0.56	0.51	0.25
MEST	0.99	0.96	0.33	0.93	0.26	0.94	0.27
H19	0.64	0.65	0.065	0.78	0.64	0.84	0.81
GRB10	0.63	0.55	0.35	0.31	0.62	**0.036***	0.46
DLK1	0.87	0.71	0.47	**0.05***	0.13	0.16	0.099
MEG3	0.622	0.262	0.700	0.165	0.696	0.939	0.913
PEG10	0.2	0.7	0.92	0.22	0.99	0.53	0.47
PEG3	0.4	0.95	**0.0019****	**0.014***	0.061	0.18	0.16
SLC22A18	0.57	0.4	0.97	0.09	0.7	0.99	0.12
CDKN1C	0.41	0.085	0.14	0.85	0.14	0.94	0.96
PLAGL1_all	0.43	0.83	0.66	0.38	0.23	0.3	0.77
PLAGL1_imp	0.4	0.83	0.39	0.64	0.78	**0.018***	0.64
IGFBP3	0.33	0.84	0.002	0.62	0.43	0.56	0.8

GA = gestational age (days), MW = maternal weight, Para = parity.

Supplementary Table 4. Primer sequences for RT-qPCR.

Gene	Sequence 5' to 3'	Size (bp)	Accession	Ref
L19	F: GCGGAAGGGTACAGCCAAT<br>R: CAGGCTGTGATACATGTGGCG	130	NM_000981	(1)
PEG10	F: TGTCTTCGCAGAGGAGTCCTCG<br>R: ACTTCTGTGGGGATGGAGGCC	105	NM_001040152	(2)
PEG3	F: TGCAGCTTGCTGAAGACGATG<br>R: AAGATTCATCTTCACAAATCCC	211	NM_006210	(2)
DLK1	F: AGCACCTATGGGGCTGAATG<br>R: TCCCAGCCGTCGGTGCAAATGC	82	NM_003836	(2)
MEG3	F: ACGGCGGAGAGCAGAGAG<br>R: AGGAGAGACCCGGGTGAG	106	NR_002766	MI
H19 (Term)	F: GGAGTTGTGGGAGACGGCCTTGAGT<br>R: CCAGTCACCCGGCCCAGATGGAG	109	NR_002196	(3)
H19 (CVS)	F: ACACTATGGCTGCCCTCTG<br>R: AGAGGGTTTTGTGTCCGGAT	100	NR_002196	MI
PHLDA2	F: GAGCGCACGGGCAAGTA<br>R: CAGCGGAAGTCGATCTCCTT	68	NM_003311	(1)
GRB10	F: CACGAGCCCTTGCTGTTTA<br>R: AAGTCCCCTCCACCCTTCTA	115	NM_001001555	(2)
IGFBP3	F: CGGTCTTCCTCCGACTCAC<br>R: GTCAACGCTAGTGCCGTCAG	91	NM_000598.4	JMF
SLC22A18	F: GCAGGATGAGCGCTCTAGGC<br>R: GCACCCCGAAGGTGGTTTGC	168	NM_183233	(2)
CDKN1C	F: CAGCTGCACTCGGGGATTTC<br>R:GATCTCTTGCGCTTGGCGAAG	125	NM_000076	(2)
PLAGL1_Imp	F: AGCCGTGCTCACAGCTCAG<br>R: AGGCAGCAGCCACATTAGAC	200	NM_001080954	II
PLAGL1_All	F: GTGAGGAGTGTGGGAAGAAG<br>R: GAGGTGGTCCAGTAGCACCTC	140	NM_001080951	II

Annealing temperature was set to 60℃ for the all qPCR primers. MI = primer designed by Miho Ishida. JMF = primer designed by Jennifer May Frost. II = primer designed by Isabel Iglesias-Platas. Imp = imprinted isoform. All = all isoforms.

Supplementary Table 5. Primer sequences for the imprinting analysis.

Gene	Sequence 5' to 3'	Tm (℃)	Polymorphism	Accession	Ref
DLK1*geno/RT	F: CACCTGCGTGGATGATGAG<br>R: GATGGTGAAGCAGATGGCCTG	58	rs1802710	NM_003836	(2)
***MEG3*geno**	F: TCACCTGCTAGCAAACTGGA<br>R: GGCTGCCTCGTTTACATACA	58	rs45617834<br>rs941575	NR_002766	(2)
***MEG3*RT**	F: AAGGACCACCTCCTCTCCAT<br>R: AGGAGGTTGCTCTCATCGAC	56			
PEG3*geno/RT	F: GAACCCTGTCAATATCTTTTTGAG<br>R: CATGAGAACCACTTCAACAAAC	57	rs1055359	NM_001146184	(2)
***ACTB*geno/RT**	F: GTCTTCCCCTCCATCGTG<br>R: GGTCATCTTCTCGCGGTTG	59	NA	NM_001101	ACT
PEG10	F: ACAGAGATGTAAGAGGCAGGC<br>R: ATTCACAGCATTGTAAGAAGTTCAC	60	rs13073<br>rs13226637	NM_015068	MI
H19	F: CTCACCCACCGCAATTCATT<br>R: CCCTGGATGCTGTACTGTCT	59	rs2067051		MI
SLC22A18	F: CCCGGATCAACTGGACTTTTG<br>R (Geno): GGCACGATGGAGAACTGCATG<br>R (RT): GCACCCCGAAGGTGGTTTGCA	58	rs1048046,<br>rs1048047	NM_183233	(4)
CDKN1C	F: CTAGCCAGCAGGCATCGAG<br>R: CTCCATCGTGGATGTGCTG	58	PAPn repeat	NM_001122631	(4)
PLAGL1	F: GAGCACCATTTAAAGGAACG<br>R: GGTTCTACCCAGACATGGACC	55	rs2076684	NM_006718_P1<br>NM_001080951_P2	II

MI = primer designed by Miho Ishida. JMF = primer designed by Jennifer May Frost. II = primer designed by Isabel Iglesias-Platas. ACT = primer designed by Anna Clare Thomas.

Reference List

(1) Apostolidou S, Abu-Amero S, O'Donoghue K, Frost J, Olafsdottir O, Chavele KM, et al. Elevated placental expression of the imprinted PHLDA2 gene is associated with low birth weight. J Mol Med (Berl) 2007 Apr;85(4):379-87.

(2) Frost JM, Monk D, Stojilkovic-Mikic T, Woodfine K, Chitty LS, Murrell A, et al. Evaluation of allelic expression of imprinted genes in adult human blood. PLoS One 2010;5(10):e13556.

(3) Gicquel C, Rossignol S, Cabrol S, Houang M, Steunou V, Barbu V, et al. Epimutation of the telomeric imprinting center region on chromosome 11p15 in Silver-Russell syndrome. Nat Genet 2005 Sep;37(9):1003-7.

(4) Monk D, Arnaud P, Apostolidou S, Hills FA, Kelsey G, Stanier P, et al. Limited evolutionary conservation of imprinting in the human placenta. Proc Natl Acad Sci U S A 2006 Apr 25;103(17):6623-8.
